# Anti-HERV-K (HML-2) capsid antibody responses in HIV elite controllers

**DOI:** 10.1186/s12977-017-0365-2

**Published:** 2017-08-22

**Authors:** Miguel de Mulder, Devi SenGupta, Steven G. Deeks, Jeffrey N. Martin, Christopher D. Pilcher, Frederick M. Hecht, Jonah B. Sacha, Douglas F. Nixon, Henri-Alexandre Michaud

**Affiliations:** 1 Department of Microbiology, Immunology, and Tropical Medicine, The George Washington University, Ross Hall 604, 2300 Eye St. NW, Washington, DC 20037 USA; 2 Gilead Sciences Inc., Foster City, CA USA; 3 Division of Experimental Medicine, Department of Medicine, University of California, San Francisco, San Francisco, CA USA; 4 HIV/AIDS Program, Department of Medicine, University of California, San Francisco, San Francisco, CA USA; 5 Department of Epidemiology and Biostatistics, University of California, San Francisco, San Francisco, CA USA; 6 Division of Pathobiology and Immunology, Oregon Health and Science University, Portland, CA USA; 7 Equipe Immunité et Cancer, Institut de Recherche en Cancérlogie de Montpellier, Montpellier, France

**Keywords:** HIV, HERV-K, Antibodies, Gag, Elite Controllers, Viremic non-controllers

## Abstract

**Background:**

Human endogenous retroviruses (HERVs) comprise approximately 8% of the human genome and while the majority are transcriptionally silent, the most recently integrated HERV, HERV-K (HML-2), remains active. During HIV infection, HERV-K (HML-2) specific mRNA transcripts and viral proteins can be detected. In this study, we aimed to understand the antibody response against HERV-K (HML-2) Gag in the context of HIV-1 infection.

**Results:**

We developed an ELISA assay using either recombinant protein or 164 redundant “15mer” HERV-K (HML-2) Gag peptides to test sera for antibody reactivity. We identified a total of eight potential HERV-K (HML-2) Gag immunogenic domains: two on the matrix (peptides 16 and 31), one on p15 (peptide 85), three on the capsid (peptides 81, 97 and 117), one on the nucleocapsid (peptide 137) and one on the QP1 protein (peptide 157). Four epitopes (peptides 16, 31, 85 and 137) were highly immunogenic. No significant differences in antibody responses were found between HIV infected participants (n = 40) and uninfected donors (n = 40) for 6 out of the 8 epitopes tested. The antibody response against nucleocapsid (peptide 137) was significantly lower (*p* < 0.001), and the response to QP1 (peptide 157) significantly higher (*p* < 0.05) in HIV-infected adults compared to uninfected individuals. Among those with HIV infection, the level of response against p15 protein (peptide 85) was significantly lower in untreated individuals controlling HIV (“elite” controllers) compared to untreated non-controllers (*p* < 0.05) and uninfected donors (*p* < 0.05). In contrast, the response against the capsid protein (epitopes 81 and 117) was significantly higher in controllers compared to uninfected donors (*p* < 0.001 and <0.05 respectively) and non-controllers (*p* < 0.01 and <0.05). Peripheral blood mononuclear cells (PBMCs) from study participants were tested for responses against HERV-K (HML-2) capsid recombinant peptide in gamma interferon (IFN-γ) enzyme immunospot (Elispot) assays. We found that the HERV-K (HML-2) Gag antibody and T cell response by Elispot were significantly correlated.

**Conclusions:**

HIV elite controllers had a strong cellular and antibody response against HERV-K (HML-2) Gag directed mainly against the Capsid region. Collectively, these data suggest that anti-HERV-K (HML-2) antibodies targeting capsid could have an immunoprotective effect in HIV infection.

**Electronic supplementary material:**

The online version of this article (doi:10.1186/s12977-017-0365-2) contains supplementary material, which is available to authorized users.

## Background

Human endogenous retroviruses (HERVs) are fossil remnants of inherited retroviruses which were endogenized into the genome, and comprise about 5–8% of the human genome [1]. Their ability to replicate or produce infectious particles is impaired by host restriction [2, 3] and they are now considered to be stably integrated, largely silent, and transmitted in a Mendelian fashion [4]. Three major HERV classes have been identified and classified according to their polymerase gene (*pol*) sequence homology with exogenous retroviruses. Class I, II and III HERVs have similarities with gammaretroviruses, betaretroviruses and spumaviruses, respectively [5]. To date, endogenous homologues to lentiviruses have not been described in the human genome.

HERV-K (HML-2), a class II HERV, with *gag*, *pro*, *pol* and *env* genes, flanked by two Long Terminal Repeats (LTR), is the most recently integrated into the genome and under certain circumstances can express proteins [6, 7]. HERV-K (HML-2) expression has been associated with some autoimmune diseases [8–13] and cancers [14–19], and mRNA transcripts and proteins can be found in tumor tissues. Translated HERV proteins can induce an immune response that correlates with disease progression or regression in some cancers [20–25].

We, and others, have previously shown that HERV-K (HML-2) can be reactivated in HIV infection [26–28]. The mechanisms leading to HERV-K (HML-2) expression are still being elucidated, but HIV Vif and Tat proteins have been implicated [27, 29]. However, it appears that the transactivation of HERV-K by exogenous HIV is more complex than initial studies suggested. In a previous study, we showed that HIV induced a skewed expression of HERV-K (HML-2) Env which favored the surface cell expression of the transmembrane envelope glycoprotein (TM) at the expense of the surface unit (SU). We showed that isolated HERV-K specific T-cell clones and HA137, a human anti-HERV-K (HML-2) TM antibody, eliminated HIV infected cells in vitro [26–28, 30, 31].

To further characterize the role of the anti-HERV-K (HML-2) immune response in HIV infection, we investigated the antibody response to HERV-K (HML-2) Gag in HIV infected participants. In this study, we showed that strong anti-HERV-K (HML-2) capsid response is more frequently found in elite controllers (ECs) compared to viremic non-controllers (VNCs) and HIV-negative low risk donors (SNLR). This response correlated with the HERV-K (HML-2) capsid T cell response. We mapped the antibody response and characterized an antibody pattern signature in ECs that significantly differed from the ones found VNCs, suggesting that the anti-HERV-K (HML-2) antibody response could play a role in the control of infection.

## Results

### The anti-HERV-K (HML-2) Capsid response correlates with anti-HERV Gag T-cell response in elite controllers

**Fig. 1:**
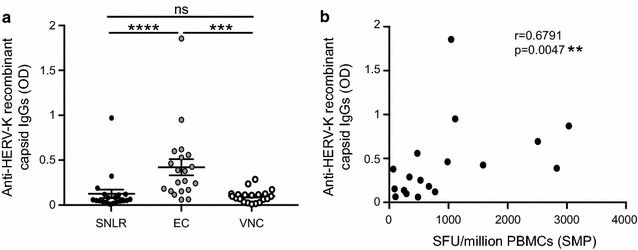
Comparison of antibody response against HERV-K (HML-2) recombinant capsid protein (**a**). Detection of antibodies against recombinant HERV-K (HML-2) capsid protein was performed by ELISA assay for 40 seronegative low risk healthy donors (SNLR) and 80 chronic HIV subjects: 40 elite controllers (EC) and 40 viremic non-controllers (VNC). *Individual dots* represent the mean of 4 independent experiments. Correlation of capsid specific T cell responses in elite controllers (**b**). Both specific T cell and antibody responses were assayed by Elispot and ELISA respectively using the recombinant HERV-K (HML-2) protein for 18 elite controllers. *Individual dots* represent the mean of 4 independent experiments for the ELISA assay. The statistical significance between the different groups was established using a Kruskal–Wallis and Dunn’s Multiple Comparison test for A and a non-parametric Spearman test for B. A *p* value <0.05 was considered as significant. *p < 0.05; **p < 0.01; ***p < 0.001; ****p < 0.0001

We first evaluated the antibody response against HERV-K (HML-2) recombinant capsid protein in uninfected donors and in untreated HIV-infected participants who were categorized as ECs or VNCs (Fig. 1). Although no significant differences were found in the magnitude of the antibody response between HIV-infected adults and HIV-negative low risk donors (SNLR), when the HIV-infected cohort was classified according to clinical status, we found that ECs had significantly higher level of antibodies against HERV-K (HML-2) capsid compared to SNLR (*p* < 0.01, Kruskal–Wallis test) and VNC (*p* < 0.001, Kruskal–Wallis test) (Fig. 1a). Since 85% of ECs developed a moderate or a strong anti-HERV Gag B-cell response (compared to 15% for VNCs), we investigated whether ECs also had a T-cell response against HERV-K (HML-2) Gag. We found that the HERV-K (HML-2) Gag antibody and T-cell response by Elispot were significantly correlated (*p* = 0.0047, Spearman test) (Fig. 1b).

### Identification of linear antibody epitopes on HERV-K (HML-2) Gag proteins

**Fig. 2:**
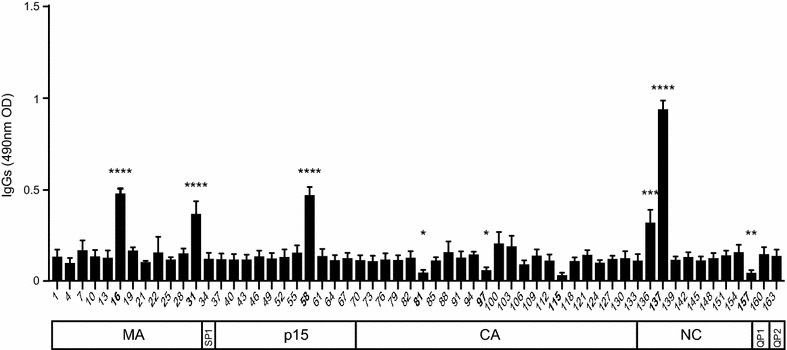
Antibody mapping of anti-HERV-K (HML-2) responses in healthy donors. Sera from 8 seronegative low risk healthy donors (SNLR) were used for antibody epitope identification by ELISA. The 7 sub-units: matrix (MA), SP1, p15, capsid (CA), nucleocapsid (NC) and QP 1 and 2 are represented by 164 redundant 15mers named by their number corresponding to their rank in the list. The lines represent the average of the 8 individuals and duplicate signals (OD). Background was determined by the average of OD from each peptide. The statistical significance between the different groups was established using a Kruskal–Wallis and Dunn’s Multiple Comparison test and a *p* value <0.05 was considered as significant. *p < 0.05; **p < 0.01; ***p < 0.001; ****p < 0.0001

To further characterize anti-HERV-K (HML-2) Gag responses during HIV infection we used a set of 164 redundant “15mer” peptides overlapping by 11 amino acids in a peptide-based ELISA assay to map immunogenic domains. We used sera from 8 SNLR and confirmed that HIV uninfected donors had a low basal level of antibodies against HERV-K (HML-2) Gag, as previously published [32]. Among all tested peptides, 8 sequences had significant differences with the basal level. We identified 4 sequences with a higher reactivity with human sera and 4 sequences associated with a lower reactivity (Table 1; Fig. 2; Additional file 1: Fig. S1). The epitopes are distributed on HERV-K (HML-2) Gag as follows: two epitopes on matrix (MA, peptides 16 and 31), one epitope on p15 (peptide 85), three epitopes on capsid (CA, peptides 81, 97 and 117), one epitope on nucleocapsid (NC, peptide 137) and one epitope on the QP1 protein (peptide 157). These results suggest that each protein domain has different antibody immunogenicity to HERV-K (HML-2) proteins (Fig. 2). Indeed, sera from SNLR participants strongly reacted to the nucleocapsid epitope but not to the capsid (Fig. 2). Thus, domains can be classified as poorly immunogenic, such as the capsid or the QP1 and QP2 proteins, or immunogenic such as matrix, p15 and nucleocapsid.

**Table 1:** Sequence identification of HERV-K (HML-2) Gag epitopes

Epitope	Sequence	Protein	Immunogenicity
16	KRIGKELKQAGRKGN	MA	Medium
31	KKSQKETESLHCEYV	MA	Medium
58	GYPGMPPAPQGRAPY	P15	Medium
81	GVKQYGPNSPYMRTL	CA	Low
97	NPPVNIDADQLLGIG	CA	Low
117	SIADEKARKVIVELM	CA	Low
137	KCYNCGQIGHLKKNC	NC	High
157	PIQPFVPQGFQGQQP	QP1	Low

*MA* matrix, *CA* capsid, *NC* nucleocapsid

### Elite controllers and viremic non-controllers have distinct antibody patterns

**Fig. 3:**
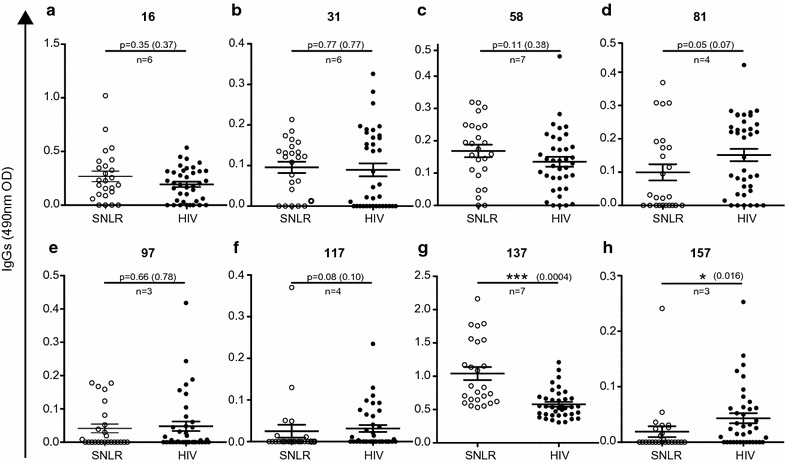
Anti-HERV-K (HML-2) antibodies in HIV infection. The detection of total IgG against HERV-K (HML-2) gag was performed for 40 seronegative low risk healthy donors (SNLR *white dots*) and 80 chronically HIV infected subjects (HIV *black dots*) by peptide-based ELISA using sequences determined in Fig. 2 and represented by their number on the top of each graph (**a**–**h**). Each graph represents ELISA for one linear epitope. *Error bars* represent SEM. The statistical significance between the different groups was established using the Mann–Whitney *u* test. The figure shows the representative results of at least three independent experiments. A *p* value <0.05 was considered as significant. *p < 0.05; **p < 0.01; ***p < 0.001. In parenthesis is indicated the adjusted *p* value (q) regarding the 3 independent experiments using original method of Benjamini and Hochberg with a Q of 5%. The number of independent observations is represented by n

We then used the eight peptide epitopes identified above to perform a serological screen on 40 HIV-infected and 40 SNLR sera samples (Fig. 3). The differences between these two groups were not significant for 6 out of 8 epitopes (Fig. 3). The responses against MA (peptides 16 and 31) and p15 (peptide 58) were either slightly decreased or not changed upon HIV infection (Fig. 3a–c). However, the response against the most immunogenic domain (peptide 137) was significantly lower (*p* < 0.001, q = 0.0004, Kruskal–Wallis test) in HIV infected subjects (Fig. 3g). Although the response against peptide 97 did not differ based on HIV status, the response against the two other epitopes present on the capsid, peptide 81 and 117, trended towards being higher in those infected with HIV (*p* = 0.05, q = 0.07 and *p* = 0.08, q = 0.1 respectively, Kruskal–Wallis test) (Fig. 3d–f). The response against peptide 157 from the QP1 protein was the only that was significantly higher among those with HIV infection (*p* < 0.05, q = 0.01 Kruskal–Wallis test) (Fig. 3h).

**Fig. 4:**
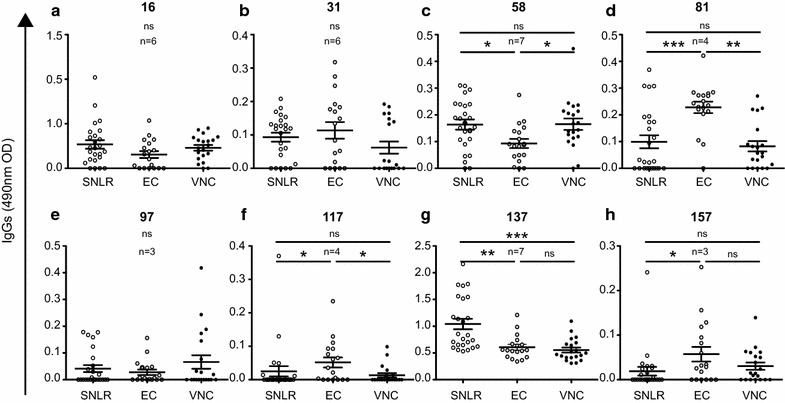
Anti-HERV-K (HML-2) capsid response in HIV infection. The detection of total IgG against HERV-K (HML-2) gag was performed for 40 seronegative low risk healthy donors (SNLR *white dots*), 40 elite controllers (EC *grey dots*) and 40 viremic non-controllers (VNC *thin black dots*) by peptide-based ELISA using sequences determined in Fig. 2. Each graph represents ELISA for one linear epitope and represented by their number on the top of each graph (**a**–**h**). *Error bars* represent SEM. The figure shows the representative results of at least three independent experiments. The statistical significance between the different groups was established using a Kruskal–Wallis and Dunn’s Multiple Comparison test and a *p* value <0.05 was considered as significant. *p < 0.05; **p < 0.01; ***p < 0.001

**Fig. 5:**
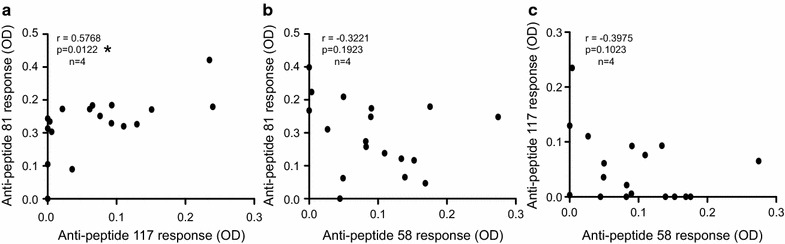
Anti-HERV-K (HML-2) response correlations. The correlation between the anti-capsid and anti-p15 responses was determined by comparing the responses against the peptides 81 and 117 for the capsid and 58 for p15 measured by peptide-based ELISA for 20 elite controllers (**a**) and 20 viremic non controllers (**b**, **c**). The statistical significance between the different responses was established using the non-parametric Spearman test. The figure shows the representative results of four independent experiments. A *p* value <0.05 was considered as significant. *p < 0.05

To better understand the potential role of these antibody responses in HIV infection, we categorized the cohort based on clinical status. We used sera samples from 20 elite controllers (ECs) and 20 viremic non-controllers (VNCs). No differences were observed for the responses against peptides 16, 31, 97, 137 and 157 (Fig. 4a, b, e, g, h). However, significant differences were observed for the response against p15 (peptide 58). The response was significantly lower in elite controllers compared to SNLR (*p* < 0.05, Kruskal–Wallis test) and VNCs (*p* < 0.05, Kruskal–Wallis test), while there was no difference between VNCs and SNLR (Fig. 4c). In contrast, antibody responses against capsid epitopes 81 and 117 were significantly higher in ECs compared to SNLR (*p* < 0.001 and <0.05, respectively, Kruskal–Wallis test) and VNCs (*p* < 0.01 and <0.05, Kruskal–Wallis test), but no differences were detected between VNCs and SNLR (Fig. 4f). We found that 90% of ECs had a moderate or a strong response against peptide 81 (25% for VNCs). Furthermore, we found a positive correlation for responses against capsid epitopes 81 and 117 (*p* = 0.0122, r = 0.5768, Spearman test) in ECs that was not found in VNCs (Fig. 5a). However, compared to the response against peptide 58, we found a trend towards an inverse relationship between the antibody responses against HERV-K (HML-2) capsid and p15 developed in VNCs. Collectively, the data show that VNCs and ECs developed different anti-HERV-K (HML-2) gag antibody responses (Fig. 5b, c).

**Fig. 6:**
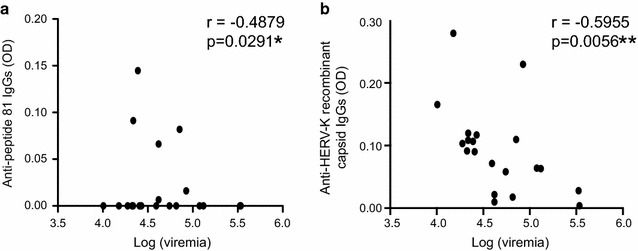
Correlations between HIV viremia and anti-HERV-K (HML-2) capsid responses. The correlation between the anti-HERV-K (HML-2) capsid response and viral load was determined by comparing the responses against peptide 81 (**a**) and the recombinant HERV-K (HML-2) capsid protein (**b**) measured by peptide-based ELISA for 20 viremic non-controllers. The statistical significance between the different responses was established using the non-parametric Spearman test. Figure 6a shows a representative result of four independent experiments. Figure 6b shows the mean of four independent experiments. A *p* value <0.05 was considered as significant. *p < 0.05; **p < 0.01

When analyzing the different groups according to their clinical status we found that VNC had low levels of anti-capsid antibodies, and there was a significant inverse correlation between the anti peptide 81 or anti-HERV-K (HML-2) recombinant capsid responses and HIV viremia (*p* = −0.4879, r = 0.0291 and *p* = 0.0056, r = −0.5955 respectively, Spearman test) (Fig. 6). However, no correlation between CD4+T cell counts and anti-HERV-K (HML-2) Gag responses were detected, either in ECs or in VNCs (data not shown).

## Discussion

We, and others, have previously shown that HIV infection reactivates HERV-K (HML-2), leading to HERV-K (HML-2) Gag and Env protein production [27, 33]. In this study, we hypothesized that HIV infection leads to a HERV-K (HML-2) capsid antibody response. We focused on responses to HERV-K (HML-2) capsid based on our previous findings that elite controllers (EC) developed an anti-HERV-K (HML-2) capsid cellular immune response [30]. We show here that a strong antibody response against HERV-K (HML-2) capsid can be detected in ECs. To better characterize the anti-HERV-K (HML-2) response, we mapped responses to linear epitopes on the full HERV-K (HML-2) Gag sequence. Responses against HERV-K (HML-2) Gag were not changed by HIV infection, except for responses to an epitope present on the QP1 protein (epitope 157), which were higher in HIV-1 infected participants compared to controls. The antibody response against nucleocapsid (epitope 137) was significantly lower in HIV infected participants compared to uninfected individuals.

When we compared the anti-HERV-K (HML-2) gag antibody response between ECs and VNCs, we saw a distinct antibody pattern, characterized by a significantly higher anti-capsid antibodies (specific to peptides 81 and 117) and lower anti-p15 antibodies (epitope 58) in controllers. The plasma HIV RNA levels were strongly inversely correlated in VNCs who had an anti-HERV-K (HML-2) capsid response.

It has been previously established that HERV-K (HML-2) expression is tightly associated with HIV viral transcription and activity in vitro and in vivo [31, 34–37]. However, ECs have limited HIV replication activity, suggesting that the induction of the anti HERV-K (HML-2) capsid response in ECs is not caused by HIV-induced HERV-K capsid expression, as has been previously described for the HERV-K (HML-2) envelope antibody response [31, 36, 37]. However, a longitudinal study would be more informative to determine potential causality.

In a previous report, we showed that expression of HERV-K Env proteins following HIV infection is skewed towards a predominant expression of the HERV-K transmembrane protein compared to the surface unit protein [31]. This suggested an HIV/HERV-K (HML-2) interaction far more complex than previous studies have proposed. In the case of HERV-K (HML-2) Gag expression, antibody profiles found in VNCs and ECs seem to reinforce this complexity. Anti-HERV-K (HML-2) capsid responses in VNCs are not significantly different than those found in controls, but they seem to be lower in early and late stages of HIV infection despite an increase of HERV-K (HML-2) expression. Patients who naturally control HIV infection are more likely to have a strong antibody response against HERV-K capsid, but the antibody response against p15 (peptide 58) was strongly decreased in ECs compared to VNCs and SNLR. Further longitudinal studies are needed to understand the chronology and the cause of this dichotomy.

A second objective of our study was to characterize the role of anti HERV-K (HML-2) capsid responses in ECs. Antibodies against viral gag proteins are not unexpected in HIV patients [38] and the anti-HIV p24 response correlates with control of disease progression [38]. Antibodies against capsid could bind cells resulting in their lysis and formation of immunocomplexes. Those immunocomplexes might interact with innate immune cells such as NK cells, macrophages or dendritic cells and promote their activation and induction of a cellular immune response [39, 40]. This may explain why both T and B cell responses directed against HIV p24 correlated with the status of controllers. A similar hypothesis could be applied for anti-HERV-K (HML-2) capsid antibodies, with EC having both an anti-HIV p24 and an anti-HERV-K (HML-2) capsid response as a way to reinforce their antiviral response.

## Conclusion

In this study, we identified linear immunogenic antibody epitopes on HERV-K (HML-2) gag proteins. We found that elite controllers had a distinctive antibody pattern compared to viremic non-controllers and HIV seronegative participants. Although further studies are needed to elucidate how these responses could be involved in the control of viremia, it reinforces the importance of studying HERV-K (HML-2) capsid immune responses in HIV infection.

## Methods

### Study populations

Samples of peripheral blood mononuclear cells (PBMCs) were selected from participants in a San Francisco-based HIV-infected cohort: OPTIONS (n = 40). Samples from HIV-negative controls were obtained from individuals who donated blood to the Stanford blood bank. Studies were performed on cryopreserved PBMCs and sera.

PBMC and sera samples were obtained from the following categories of chronically HIV-infected individuals: 20 elite controllers (EC: naive for treatment, undetectable viral load for two years, CD4 > 350) and 20 untreated virologic non-controllers (VNC; naive for treatment, viral load >2000 copies/mL).

### ELISA

A set of 164 overlapping “15-mer” HERV-K (HML-2) Gag peptides (JPT Peptide Technologies, Berlin, Germany), based on HERV-K102 sequence (AF164610), were used to comprehensively map the HERV-K (HML-2) antibody response (Additional file 2: Fig. S2). Positive signals were confirmed by peptides produced by two other companies (New England Peptide and Gene Script). 96 microtiter wells plate (Nunc-Immuno Plate MaxiSorp Surface) were coated for 1 h at 37 °C with peptides at 10 μg/ml in PBS or over-night at 4 °C with recombinant protein (GeneArt) at 5 μg/ml in PBS. Plates were then washed 3 times with 200 μL of PBS/0.05%-Tween 20 and blocked with 100 μL of blocking buffer (PBS/2.5%-BSA) at room temperature (RT). The samples were diluted in blocking buffer and incubated 2 h at RT in duplicates. Plates were then washed 3 times with 200 μL of PBS/0.05%-Tween 20. An anti-human IgG or anti-human IgM HRP-conjugated secondary antibody was diluted at 1:1000 in blocking buffer and incubated at RT for 1 h. Plates were then washed 6 times with 200 μL of PBS/0.05%-Tween 20 and incubated for 10 min with 100 μL of TMB (Invitrogen). Addition of 50 μL H2SO4 2 M stopped the reaction. The plates were read at 450 and 690 nm for the background on a plate reader. Background from 690 nm uncoated wells and twice the background from 450 nm PBS-BSA (negative control) were subtracted from the mean absorbance of the coated wells (corrected OD). For the detection of anti-Gag antibodies, sera were used at 1:400. ODs were normalized with serum from a high responder in a standard curve. The STDEV intra experiment was less than 4%.

For the response against the recombinant HERV-K (HML-2) capsid and the peptide 81, we defined the humoral responses for elite controllers as followed: the response was considered moderate if the corrected OD is greater than the mean of corrected OD for SNLR and strong if the corrected OD is greater than twice the mean of corrected OD for SNLR.

### ELISPOT assays

The ELISPOT assay has been described previously [30]. In brief, 96-well plates (Millipore, Billerica, MA) were coated with human monoclonal anti-interferon gamma (IFN-γ) immunoglobulin (Mabtech, Mariemont, OH). After plates were washed and blocked with 10% fetal calf serum, PBMCs were added at a concentration of 10^5^ cells per well. Duplicate wells were prepared for each experimental condition. Spot totals for duplicate wells were averaged, and all spot numbers were normalized to numbers of (IFN-γ) spot forming units (SFU) per million PBMCs (SPM). The spot values from medium control wells were subtracted, after which a positive response to a peptide was defined as 50 SPM and 2 times the medium control value. The total magnitude of the HERV T cell response was calculated by adding up all of the individual peptide SPM values.

### Statistical analyses

To assess the distribution of the humoral responses obtained in this study, we used the D’Agostino and Pearson normality test. The results concluded that the populations were not normally distributed. According to this statement, we used non-parametric statistical tests to compare the humoral responses assayed by ELISA for each group. Multiple comparisons were performed in the 3 groups (SNLR, EC and VNC) with the Kruskal–Wallis and Dunn’s multiple comparison test for Fig. 4. Spearman correlation analyses were used to measure associations between different humoral responses and HIV viral load or CD4+T cells count for Figs. 1b, 5, and 6. The two-tailed Mann–Whitney *u* test was used to compare the humoral responses between HIV-1pos and HIV-1neg (SNLR) groups for Fig. 3. A *q* value was calculated using the original method of Benjamini and Hochberg and added on the figure when it was relevant. All tests were conducted using GraphPad Prism, version 6.00 (GraphPad Software, San Diego, CA), with the statistical significance of the findings set at a *p* value of less than 0.05.

## Additional files

**Additional file 1: Fig. S1.** Antibody mapping of anti-HERV-K (HML-2) responses in study participants groups. Sera from Healthy donors, Elite Controllers and Viremic non-controllers were used for antibody epitope identification by ELISA. The 7 sub-units: matrix (MA), SP1, p15, capsid (CA), nucleocapsid (NC) and QP 1 and 2 are represented by 164 redundant 15mers named by their number corresponding to their rank in the list. The lines represent the average of the 8 individuals and duplicate signals (OD). Background was determined by the average of OD from each peptide. Peptides giving a signal significantly decreased are symbolized in grey and peptides giving a signal significantly increased are symbolized in black.

**Additional file 2: Fig. S2.** Strategy for designing overlapping peptides.
