# Supplementary material for: Anti-HERV-K (HML-2) capsid antibody responses in HIV elite controllers
Source: Retrovirology. 2017 Aug 22;14:41. doi: 10.1186/s12977-017-0365-2 (PMC5568399; doi:10.1186/s12977-017-0365-2)
Supplement: Supplementary file 2 — Additional file 2: Fig. S2. Strategy for designing overlapping peptides. [file 12977_2017_365_MOESM2_ESM.pdf]

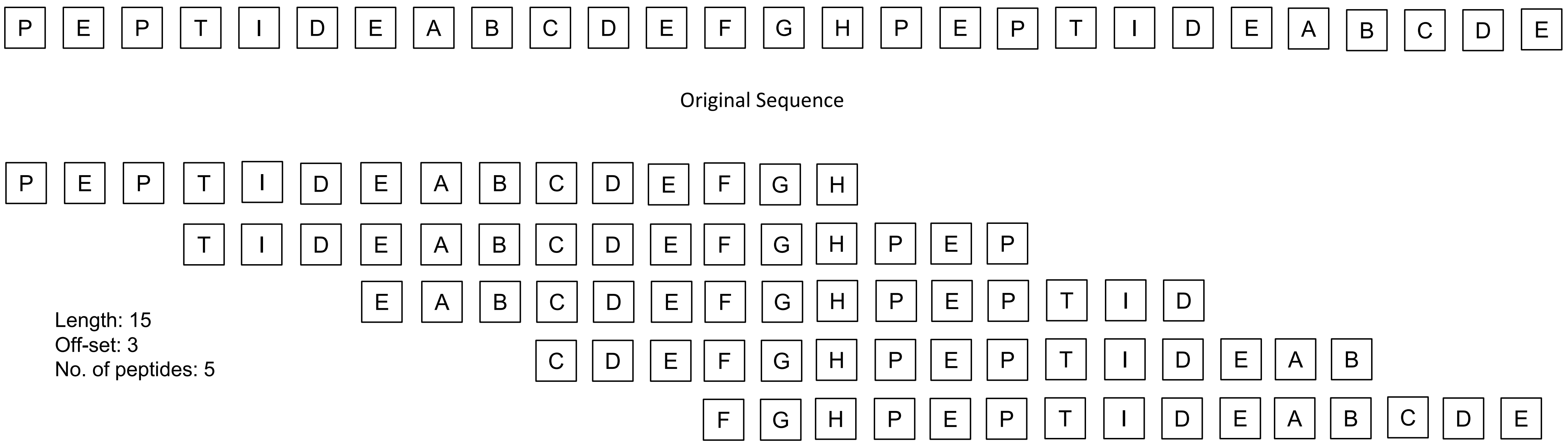
P E P T I D E A B C D E F G H P E P T I D E A B C D E
Original Sequence
P E P T I D E A B C D E F G H
T I D E A B C D E F G H P E P
E A B C D E F G H P E P T I D
C D E F G H P E P T I D E A B
F G H P E P T I D E A B C D E
Length: 15
Off-set: 3
No. of peptides: 5
